# Management of acute appendicitis during COVID-19 pandemic. Single center data from a tertiary care hospital in Germany

**DOI:** 10.1515/iss-2022-0021

**Published:** 2023-11-13

**Authors:** Mihailo Andric, Jessica Stockheim, Mirhasan Rahimli, Michael Klös, Torben Esser, Ivan Soldatovic, Maximilian Dölling, Sara Al-Madhi, Sara Acciuffi, Roland Croner, Aristotelis Perrakis

**Affiliations:** Department of General, Visceral, Vascular and Transplant Surgery, University Hospital Magdeburg, Magdeburg, Germany; Institute of Microbiology and Hospital Hygiene, University Hospital Magdeburg, Magdeburg, Germany; Institute for Medical statistics, Faculty of Medicine, University Belgrade, Belgrade, Serbia

**Keywords:** acute appendicitis, appendicectomy, conservative treatment, COVID-19

## Abstract

**Objectives:**

The unexpected global overload of the health system during COVID-19 pandemic has caused changes in management of acute appendicitis worldwide. Whereas conservative treatment was widely recommended, the appendicectomy remained standard therapy in Germany. We aimed to investigate the impact of COVID-19 pandemic on treatment routine for acute appendicitis at University Hospital of Magdeburg.

**Methods:**

Adult patients with clinical and/or radiological diagnosis of acute appendicitis were included in the single center retrospective study. Data was collected to patient demographics, treatment modality and outcomes including morbidity and length of stay. The patient data related to COVID-19 period from March 22, 2020 to December 31, 2021 (649 days) were compared to the Non-COVID-19 period from June 12, 2018 to March 21, 2020 (649 days). Subgroup analysis related to conservative or surgical treatment has been performed.

**Results:**

A total of 385 patients was included in the study, 203 (52.73 %) during Non-COVID-19 period and 182 (47.27 %) during COVID-19 period. Mean age of entire collective was 43.28 years, containing 43.9 % female patients (p=0.095). Conservative treatment was accomplished in 49 patients (12.7 % of entire collective), increasing from 9.9 % to 15.9 % during COVID-19 period (p=0.074). Laparoscopic appendicectomy was performed in 99.3 % (n=152) of operated patients during COVID-19 period (p=0.013), followed by less postoperative complications compared to reference period (23.5 % vs. 13.1 %, p=0.015). The initiation of antibiotic therapy after the diagnosis increased from 37.9 % to 53.3 % (p=0.002) during COVID-19 period regardless the following treatment modality. Antibiotic treatment showed shorter duration during pandemic period (5.57 days vs. 3.16 days, p<0.001) and it was given longer in the conservative treatment group (5.63 days vs. 4.26 days, p=0.02). The overall length of stay was shorter during COVID-19 period (4.67 days vs. 4.12 days, p=0.052) and in the conservative treatment group (3.08 days vs. 4.47 days, p<0.001). However, the overall morbidity was lower during the COVID-19 period than before (17.2 % vs. 7.7 %, p=0.005) and for conservative therapy compared to appendicectomy (2 % vs. 14.3 %, p=0.016). There was no mortality documented.

**Conclusions:**

According to our findings the COVID-19 pandemic had a relevant impact on treatment of acute appendicitis, but it was possible to maintain the traditional diagnostic and treatment pathway. Although laparoscopic appendicectomy remains a recommended procedure, the conservative treatment of uncomplicated appendicitis with excellent short-term outcome can be a safe alternative to surgery during potential new wave of COVID-19 pandemic and in the daily routine.

## Introduction

After the outbreak of pneumonia of unknown etiology in December of 2019 in Wuhan City, Hubei Province in China, the World Health Organization (WHO) identified the novel Severe Acute Respiratory Syndrome Coronavirus 2 (SARS-CoV-2) as causal and named the new disease COVID-19 [1]. In following COVID-19 reached pandemic status and caused an international health crisis [1]. Due to acute need for hospitalization and intensive care of rising number of COVID-19 patients, many medical disciplines, including surgery, were strongly affected. Numerous elective surgeries have been postponed or even cancelled [2], [3], [4].

The lack of clinical experiences and scientific data set a dilemma, if patients infected with SARS-CoV-2 would meet an impaired postoperative course, considering higher morbidity and mortality after a surgery. Additionally, it was unclear if admission of infected patients would endanger already hospitalized uninfected surgical patients and medical staff [3, 5, 6].

Therefore, several recommendations for clinical management during the COVID-19 pandemic have been published by surgical societies [7, 8]. The European Society of Trauma and Emergency Surgery (ESTES) recommended postponing of all elective surgeries during COVID-19 pandemics [9]. The treatment of emergencies such as acute appendicitis became also challenging, especially due to non-operative concepts with antibiotics being introduced in last years [10, 11]. Diverse studies showed success of non-operative management for uncomplicated acute appendicitis in over 90 % of cases for initial treatment course, 80 % after 3 months and 73–80 % after one year [11], [12], [13], [14], [15]. The recurrence of acute appendicitis is still being an issue possibly reaching a 5-year rate of 39 % according to APPAC-Study [16]. Although some risk factors for failure of non-operative treatment of appendicitis have been identified such as complicated appendicitis, faecolith, diabetes etc., the forecast of disease recurrence is still not reliable [10, 17, 18]. However, patients who needed an appendicectomy after experiencing failure of conservative treatment did not show higher morbidity than primarily operated patients [12, 14, 17]. The first line therapy with antibiotics obviously becomes an alternative to emergent appendectomy for uncomplicated appendicitis [10].

Now, in the pandemic situation the treatment concepts for acute appendicitis have been challenged for medical, epidemiological and organizational reasons. Therefore, the first line therapy with antibiotics for uncomplicated acute appendicitis in the COVID-19 pandemic has been recommended in UK and USA [8, 19, 20]. At the same time, laparoscopic appendicectomy remained the recommended standard for acute appendicitis in Germany - even during COVID-19 pandemic [21].

The first national lockdown in Germany with extensive contact restrictions was declared on March 22, 2020 [22]. In our center, elective surgery capacity during the COVID-19 period had to be reduced to 75 %, whereas only oncological indications and highly symptomatic benign diseases were allowed to be scheduled. On the other hand, the emergency program has been continuously performed. Therefore, in the case of justified suspicion on acute appendicitis, an indication for laparoscopic appendectomy was unquestionably accepted by anesthesia. Still, in selected cases conservative treatment for suspected uncomplicated appendicitis was initiated after informed consent.

The aim of this study was to investigate the impact of the COVID-19 pandemic on treatment routine and outcomes of acute appendicitis at a tertiary care hospital in Germany. For this purpose, we investigated adult patients with appendicitis between March 22 2020 and December 31, 2021 (649 days). The comparison group was defined as “Non-COVID-19” and consisted of patients treated during the same time period (649 days) before COVID-19 pandemic.

## Patients and methods

### Patients

A total of 385 adult patients (at least 18 years old) who underwent appendectomy or conservative (non-operative) treatment for acute appendicitis at the University Hospital Magdeburg in the period June 12, 2018 to December 31, 2021 were included in the study.

Children (under 18 years old) and patients who underwent simultaneous appendectomy during other surgeries were excluded.

Patients meeting inclusion criteria were assigned to two groups: 182 patients which required treatment for acute appendicitis during the **COVI** **D** **-** **19 **period from March 22, 2020 to December 31, 2021 (649 days) and 203 patients during the **Non-COVI** **D** **-** **19 **period from June 12, 2018 to March 21, 2020 period (649 days).

**COVI** **D** **-** **19** cohort included 153 patients who underwent surgical treatment and 29 patients with conservative management of acute appendicitis. **Non-COVI** **D** **-** **19** cohort included 183 patients who were treated surgically and 20 patients with conservative treatment of acute appendicitis.

### Definitions

The classification of acute appendicitis depending on inflammation grade occurred according to EAES 2015 recommendations in uncomplicated appendicitis (isolated inflammation of appendix vermiformis without appendix wall defect or surrounding reaction) and complicated appendicitis (phlegmon, gangrene, abscess, perforation) [23].

Conservative (non-operative) treatment is considered as treatment with antibiotics and/or symptomatic medication.

Failure of conservative treatment means missing improvement of symptoms and inflammation signs during 24 h of therapy with antibiotics with consequence of performing an appendicectomy.

All postoperative complications or complications during non-operative management were defined as overall morbidity during the period of 30 days of follow up.

The postoperative length of stay (pLOS) implies the duration of postoperative hospitalization measured in days for surgical patient group.

The overall length of stay (oLOS) means the time from admission to the ward until discharge for entire study collective and includes preoperative treatment period additionally to postoperative period for surgical treatment group.

The readmission rate is considered the rate of in-house readmission within 3 months after the initial treatment.

### Statistical analysis

The patient data have been acquired and analyzed retrospectively. The data regarding patient characteristics, the whole diagnostic and treatment course including treatment outcomes have been compared between the COVID-19 and Non-COVID-19 period. A subgroup analyzes according to the treatment approach (surgical or conservative treatment) have been performed.

All data analysis was performed with IBM SPSS Statistics for Windows, Version 28 (IBM Corp., Armonk, NY, USA). Depending on the type of variable we applied the Mann–Whitney-U test or T-test, as well as Pearson’s chi-squared test or Fisher’s exact test for comparison of two groups. For data presentation we used the mean and standard deviation (SD) or the number of cases with percentages in accordance with the type of data. P-values of <0.05 were considered statistically significant.

## Results

### Therapy concept during COVID-19 pandemic

During the COVID-19 period, the overall approach for treatment of acute appendicitis at the University Hospital Magdeburg has not changed. At our institution, the standard procedure for treatment of acute appendicitis in adults is laparoscopic appendicectomy. According to international literature, patients with mild clinical presentation and radiological suspicion of uncomplicated appendicitis required informed consent and have individually been offered a first line treatment with antibiotics [24]. If antibiotic therapy failed, an appendectomy has been performed.

### Frequency of appendicitis cases

**Figure 1:**
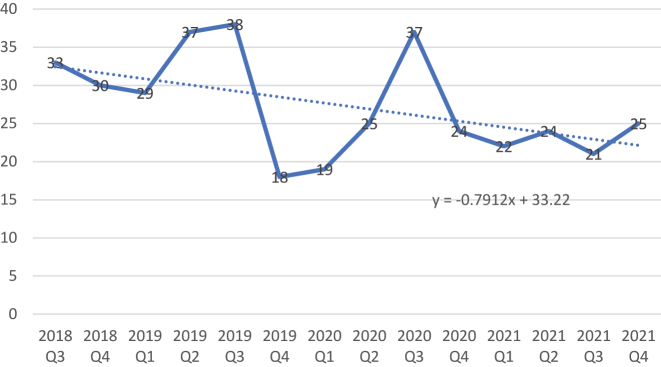
Presentation of appendicitis case frequency related to year quarters (Q3 2018–Q4 2021).

Regarding the frequency of appendicitis cases, we observed continuous reduction of average case number throughout investigated period from June 12, 2018 to December 31, 2021, as presented in Figure 1. With beginning of COVID-19 period there has been an obvious reduction of presented cases, followed by high peak during third quarter of the year 2020. This observation was not significant (p=0.142).

### Patient characteristics

Patient characteristics are presented in Table 1. The whole collective contains 169 female (43.9 %) and 216 male (56.1 %) patients. The percentage of male was higher in both examined periods without significant difference (p=0.095). The average age of entire collective was 43.28 years; in the COVID-19 cohort 42.72 years and in non-COVID-19 cohort 43.78 years without relevant difference (p=0.569). The stratification of patients according to age groups also showed no difference between the examined periods (p=0.909).

**Table 1:** Presentation of patient characteristics of the entire collective, including surgical and conservative treatment.

Patient characteristics		Non-COVID-19		COVID-19		
		n or mean	% or SD	n or mean	% or SD	
Number of patients		203	52.73 %	182	47.27 %	
SARS-CoV-2 positive	Yes	/	/	0	0 %	
	No	/	/	0	0 %	
Sex	Male	122	60.1 %	94	51.6 %	0.095
	Female	81	39.9 %	88	48.4 %	
Age	(years)	43.78	18.64	42.72	17.68 %	0.569
Age intervals	18–29	65	32.0 %	49	26.9 %	0.909
	30–49	57	28.1 %	68	37.4 %	
	50–69	59	29.1 %	49	26.9 %	
	≥70	22	10.8 %	16	8.8 %	
BMI	(kg/m^2^)	26.67	5.19	27.47	6.41	0.181
Adipositas	Yes	160	80.0 %	134	73.6 %	0.140
	No	40	20.0 %	48	26.4 %	
Diabetes	Yes	16	7.9 %	9	4.9 %	0.243
	No	187	92.1 %	173	95.1 %	
	ASA I	60	29.6 %	66	36.3 %	
ASA-score	ASA II	115	56.7 %	90	49.5 %	0.359
	ASA III	27	13.3 %	26	14.3 %	
	ASA IV	1	0.5 %	0	0 %	

Furthermore, there was no significant difference between the appendicitis patients during COVID-19 and Non-COVID-19 period in terms of BMI, obesity, diabetes and American Society of Anesthesiologists (ASA) score.

### Pretreatment parameters

Pretreatment parameters of all included patients are shown in Table 2.

**Table 2:** Presentation of pretreatment parameters of the entire collective, including surgical and conservative treatment.

Pretreatment parameters		Non-COVID-19		COVID-19		p-value
		n or mean	% or SD	n or mean	% or SD	
Number of patients		203	52.73 %	182	47.27 %	
Symptom duration	<6 h	26	13.1 %	23	12.8 %	0.858
	6h–12 h	25	12.6 %	25	14.0 %	
	12h–24 h	54	27.1 %	42	23.5 %	
	>24 h	94	47.2 %	89	49.7 %	
Leukocytes	(Gpt/l)	13.91	4.76	14.05	4.82	0.771
CrP	(mg/l)	60.77	78.33	60.92	72.69	0.857
CrP intervals	<5 mg/L	38	18.7 %	39	21.4 %	0.102
	5–49.9 mg/L	93	45.8 %	64	35.2 %	
	>50 mg/L	72	35.5 %	79	43.4 %	
Ultrasound	No	24	11.8 %	27	14.8 %	0.384
	Yes	179	88.2 %	155	85.2 %	
CT	No	157	77.3 %	127	69.8 %	0.092
	Yes	46	22.7 %	55	30.2 %	
Admission until diagnosis	(min)	448.16	1,685.69	339.38	888.49	0.374
Diagnosis until surgery	(min)	380.78	504.08	356.26	365.49	0.790
Admission until surgery	(min)	828.94	1,766.25	695.64	995.40	0.538

After admission in the emergency room all patients underwent a SARS-CoV-2 swab test and the whole examined cohort was tested negative.

All the patients obtained routine blood analysis including inflammation markers. There was no significant difference in mean values of leukocytes and CrP between Non-COVID-19 and COVID-19 cohort (13.91 Gpt/l vs. 14.05 Gpt/l, p=0.771 and 60.77 mg/L vs. 60.92 mg/L, p=0.857) respectively. However, the stratification of CrP values in intervals showed rising number of patients with CrP>50 mg/L during pandemic period. This effect was not substantial for entire collective (p=0.102), but it was statistically relevant in the subgroup analysis of operated patients (p=0.042). Normal CrP-values before and during COVID-19 were documented in around 20 % of patients before and during COVID-19.

The analysis has shown no difference in duration of symptoms until patients with appendicitis referred to our hospital during the COVID-19 pandemic and during the control period (p=0.858). Among around half of all patients, symptoms lasted more than 24 h before seeking the emergency room.

Furthermore, there was no difference during the COVID-19 and Non-COVID-19 period regarding time spent either from admission to diagnosis of acute appendicitis or from the moment of diagnosis until surgery, as well as from admission in emergency room until begin of surgical treatment (p=0.374; p=0.538; p=0.790) respectively.

We registered radiological diagnostics in terms of ultrasound and/or CT in nearly all included patients (99.7 %). The analysis showed less ultrasound examinations (88.2 % vs. 85.2 %) and more CT scans (22.7 % vs. 30.2 %) being performed during COVID-19 (p=0.384, p=0.092). Only one patient during pandemic period underwent MRI of abdomen for securing the diagnosis.

### Intraoperative parameters

The intraoperative aspects regarding surgical cohort are displayed in Table 3. A total of 336 patients underwent appendectomy for acute appendicitis, from which 323 patients (96.13 %) had laparoscopic appendectomy, 3 patients (0.89 %) patients had a primary open appendectomy and 10 patients (2.97 %) needed conversion from laparoscopy to open approach. The rate of laparoscopic appendectomy increased throughout COVID-19 pandemic from 93.4 to 99.3 % (p=0.013). The duration of appendectomy remained unchanged during pandemic (p=0.208).

**Table 3:** Presentation of intraoperative characteristics of the surgical treatment group.

Intraoperative parameters			Non-COVID-19		COVID-19		p-value
			n or mean	% or SD	n or mean	% or SD	
Number of patients			183	54.46 %	153	45.54 %	
Surgical approach	Laparoscopy		171	93.4 %	152	99.3 %	0.013
	Laparotomy		3	1.6 %	0	0.0 %	
	Conversion		9	4.9 %	1	0.7 %	
Operateur qualification	Resident		91	49.7 %	85	55.6 %	0.456
	Fellow		69	37.7 %	54	35.3 %	
	Consultant		23	12.6 %	14	9.2 %	
Duration of surgery	(min)		59.2	23.6	62.8	27.7	0.208
Complications (intraoperative)	Yes		12	6.6 %	4	2.6 %	0.091
	No		171	93.4 %	149	97.4 %	
Classification of acute appendicitis	Uncomplicated		74	40.4 %	48	31.4 %	0.085
	Complicated		109	59.6 %	105	68.6 %	
Subgroups	Uncomplicated		74	40.4 %	48	31.4 %	0.216
	Complicated	Phlegmon	49	26.8 %	52	34.0 %	
		Gangrene	16	8.7 %	21	13.7 %	
		Abscess	9	4.9 %	8	5.2 %	
		Perforation	35	19.1 %	24	15.7 %	

All the cases of acute appendicitis have been classified depending on intraoperative finding according to the recommendation EAES 2015 [23]. During the COVID-19 pandemic the proportion of complicated appendicitis increased from 59.6 to 68.6 %. This dynamic was not significant (p=0.091).

Although the increase of patients with periappendiceal phlegmon or appendix gangrene was documented, this difference between two investigated periods was insignificant (p=0.085). The stratification of cases with complicated appendicitis in subgroups showed no difference between COVID-19 and non-COVID-19 period neither (p=0.216).

As far as the appearance of intraoperative complications (such as bleeding, primary appendix stump insufficiency, lesions during adhesiolysis) are concerned, there was a trend in favour of COVID-19 collective, although without statistical significance (6.6 % vs. 2.6 %, p=0.091).

### Postoperative outcomes

The postoperative outcomes of patients after an appendicectomy are presented in Table 4 *.*

**Table 4:** Presentation of postoperative outcomes of patients who underwent an appendicectomy.

Postoperative outcomes		Non-COVID-19		COVID-19		p-value
		n or mean	% or SD	n or mean	% or SD	
Number of patients		183	54.46 %	153	45.54 %	
pLOS	(days)	4.4	2.9	3.9	2.3	0.084
Postoperative complications	Yes	34	18.6 %	14	9.2 %	0.014
	No	149	81.4 %	139	90.8 %	
Postoperative complications (type)	Surgical	14	7.7 %	6	3.9 %	0.048
	Non-surgical	20	10.9 %	8	5.2 %	
	No complications	149	81.4 %	139	90.8 %	
Clavien Dindo	I–II	24	13.1 %	8	5.2 %	0.028
	III–IVa	11	6.0 %	6	3.9 %	
	No	148	80.9 %	139	90.8 %	
Wound infection	Yes	7	3.8 %	1	0.7 %	0.076
	No	176	96.2 %	152	99.3 %	
Wound bleeding	Yes	2	1.1 %	3	2.0 %	0.663
	No	181	98.9 %	150	98.0 %	
Intraabdominal bleeding	Yes	1	0.5 %	1	0.7 %	1.000
	No	182	99.5 %	152	99.3 %	
Intrabdominal abscess	Yes	3	1.6 %	2	1.3 %	1.000
	No	180	98.4 %	151	98.7 %	
Passage delay	Yes	10	5.5 %	1	0.7 %	0.014
	No	173	94.5 %	152	99.3 %	
Passage delay expression	Constipation	5	2.7 %	0	0.0 %	0.041
	Ileus	5	2.7 %	1	0.7 %	
	No	173	94.5 %	152	99.3 %	

Regarding the postoperative length of stay there was no significant difference between the two collectives, although it was slightly shorter during COVID-19 (4.4 days vs. 3.9 days, p=0.084).

The postoperative morbidity within 30 days of follow up was documented in 14 patients (9.2 %) during the pandemic, compared to 34 patients (18.6 %) before COVID-19 (p=0.014). Of those, there have been 6 patients (3.9 %) with surgical complication and 8 patients (5.2 %) with non-surgical complication during COVID-19 period. On the othe hand, during Non-COVID-19 period, 14 patients (7.7 %) with surgical and 20 patients (10.9 %) with non-surgical morbidity have been documented, significantyl more than within COVID-9 collective (p=0.048).

In terms of Clavien-Dindo-classification there have been more postoperative complications of all classes documented during the Non-COVID-19 period than during the pandemic period (p=0.054). In comparison of periods before COVID-19 and during COVID-19, the group Clavien-Dindo I-II implied 24 (13.1 %) vs. 8 patients (5.2 %) and the group Clavien-Dindo III-IVa included 11 (6.0 %) vs. 6 (3.9 %) patients. No patients developed complications of Clavien-Dindo classes IVb and V.

The proportion of most usual complications as wound infection, wound bleeding, intraabdominal bleeding, and intraabdominal abscess were similar between both cohorts. Delayed postoperative passage, both postoperative constipation and ileus, appeared significantly less common in the pandemic cohort than in the reference cohort (p=0.014; p=0.041).

### Outcomes of conservative treatment

The characteristics of patients’ outcome after conservative treatment of acute appendicitis related to comparison between COVID-19 and non-COVID-19 period are presented in Table 5. The outcomes related to comparison between conservative and surgical treatment are presented in Table 6.

**Table 5:** Presentation of characteristics of conservative treatment related to COVID-19 and non-COVID-19 periods.

Conservative treatment I		Non-COVID-19		COVID-19		p-value
		n or mean	% or SD	n or mean	% or SD	
Number of patients		203	52.73 %	182	47.27 %	
Therapy	Conservative	20	9.9 %	29	15.9 %	0.074
	Surgery	183	90.1 %	153	84.1 %	
Antibiotics initially	Yes	77	37.9 %	97	53.3 %	0.002
	No	126	62.15 %	85	46.7 %	
Duration of antibiotic therapy	(days)	5.57	3.814	3.16	3.511	<0.001
Failure of conservative treatment (>24 h)	Yes	4	2.0 %	2	1.1 %	0.688
	No	199	98.0 %	180	98.9 %	
oLOS	(days)	4.67	3.03	4.12	2.54	0.052
Morbidity (overall)	Yes	35	17.2 %	14	7.7 %	0.005
	No	168	82.8 %	168	92.3 %	
Readmissions (3 months)	Yes	3	1.5 %	2	1.1 %	0.551
	No	200	98.5 %	180	98.9 %	

**Table 6:** Presentation of characteristics of outcome related to conservative or surgical treatment.

Conservative treatment II		Conservative		Surgery		p-value
		n or mean	% or SD	n or mean	% or SD	
Number of patients		49	12.7 %	336	87.27 %	
Antibiotics initially	Yes	47	95.9 %	127	37.8 %	<0.001
	No	2	4.1 %	209	62.2 %	
Duration of antibiotic therapy	(days)	5.63	2.45	4.26	3.99	0.02
oLOS	(days)	3.08	2.25	4.47	2.53	<0.001
Morbidity (overall)	Yes	1	2.0 %	48	14.3 %	0.016
	No	48	98 %	288	85.7 %	
Readmissions (3 months)	Yes	3	6.1 %	2	0.6 %	0.001
	No	46	93.9 %	334	99.4 %	

A total of 49 patients (12.7 % of entire collective) underwent successful initial conservative treatment of acute appendicitis. Additional 6 patients (1.6 % of the entire collective), of which 2 patients during pandemic and 4 patients before pandemic (p=0.688), underwent an appendicectomy after an insufficient conservative treatment with antibiotics for at least 24 h. These patients were included to the surgical group.

The proportion of patients treated conservatively increased from 9.9 to 15.9 % during COVID-19 period (p=0.074).

The number of patients who received antibiotics immediately after diagnosis of acute appendicitis (including patients with conservative treatment and patients with antibiotics before attended surgery) increased from 37.9 to 53.3 % (p=0.002). Antibiotics have been initiated in 95.9 % of conservatively treated patients and in 37.8 % of patients before they underwent appendectomy (p<0.001). The average duration of antibiotic therapy was longer before the pandemic (5.57 days vs. 3.16 days, p<0.001) and it was significantly longer in the conservative treatment group (5.63 days vs. 4.26 days, p=0.02).

The overall length of stay was shorter during COVID-19 period then during the reference period (4.67 days vs. 4.12 days, p=0.052). It was also relevantly shorter in the conservative than in the surgical treatment group (3.08 days vs. 4.47 days, p<0.001).

The overall morbidity during 30 days of follow up was recorded in 35 patients (17.2 %) for the Non-COVID-19 period and in 14 patients (7.7 %) for the COVID-19 period (p=0.005). Significantly more complications were observed after surgical treatment than for non-operative management of acute appendicitis (14.3% vs. 2.0 %, p=0.016). There was no mortality of appendicitis patients registered throughout the study period.

The 3-months readmission rate (mostly recurrent disease for conservative group or local intraabdominal inflammation and wound issues for surgical group) recorded after discharge from initial treatment was not different related to Non-COVID-19 and COVID-19 periods (1.5 % vs. 1.1 %, p=0.551), but it was significantly higher for the conservatively treated than surgically treated group of patients (6.1 % vs. 0.6 %, p=0.001).

## Discussion

The treatment of acute appendicitis was intensively investigated in the last years [23, 24]. To apply tailored approach, a stricter differentiation of inflammation grade of appendicitis with the new classification by EAES has been proposed [23]. A paradigm shift towards conservative treatment of uncomplicated appendicitis as an alternative to emergent appendicectomy was reported internationally [24]. The unexpected global overload of the health system during COVID-19 pandemic has forced changes in routine treatment of acute appendicitis worldwide [20].

Regarding to the frequency of appendicitis cases referring to our hospital, we observed continuous reduction of average case number through the study period (June 12, 2018 to December 31, 2021), however without significance (p=0.142). As presented in the results (Figure 1), there was an abrupt reduction of appendicitis cases at the beginning of COVID-19 period, which overlaps with overall rising number of COVID-19 cases from April to June 2020 in Germany [25]. Furthermore, we observed a peak of appendicitis cases during third quartal of the year 2020. This finding might be the result of increase of the incidence according to the disease seasonality [26].

In the present study similar baseline characteristics of the appendicitis patients during COVID-19 and Non-COVID-19 period have been shown. However, more patients showed relevantly elevated inflammatory signs, as far as CrP is concerned, with values over 50 mg/L in the surgical group during the pandemic period (p=0.042), indicating a complicated appendicitis [24, 27, 28].

Indeed, more CT scans have been performed during the pandemic than before (22.7 % vs. 30.2 %, p=0.092) also in comparison to other centers in Germany before COVID-19 pandemic (19.9 % in 2017 according to Schildberg et al.) [29], but the rate of 70 % as reported in the UK during COVID-19 lockdown was never reached [11, 30].

The internationally reported increase of complicated appendicitis rates during the pandemic period [31] was intraoperatively confirmed in our institution (59.6–68.6 %). However, probably due to relatively small case series, this observation was not statistically relevant (p=0.085).

A possible explanation for this phenomenon could be the patient’s hesitation to consult their doctor, out of fear of SARS-CoV-2 infection, as already discussed in other studies [3, 32]. These concerns were repeatedly communicated by patients in the present collective throughout anamnesis. However, this hypothesis is not supported by the available results, which shown no difference in symptom duration before approaching the emergency room between two examined periods, in contrast to data reported by Willms et al. [32].

According to our previous data, the laparoscopic approach for appendicectomy was evolving to standard at our institution with proportion of 33.1 % in years 1996/97 to 85.8 % in years 2008/09 [33], finally reaching 99.3 % in present study during the COVID-19 period, significantly more than during the interval before COVID-19 (93.4 %, p=0.013). This fact jointed with tendentially less intraoperative morbidity such as bleeding, small intestine lesions etc. and higher use of antibiotics during pandemic (p=0.002), could be an explanation for significantly less postoperative complications during the pandemic then in the reference period (p=0.015; p=0.013). The above-mentioned study also recorded less complications (p>0.001) related to increase of laparoscopic approach for appendicectomy [33]. Although increasing numbers of open appendicectomies during COVID-19 pandemic are reported from other German centers [34], we did not share the same experience. The higher rate of antibiotics administered during the COVID-19 period could be a result of successive implementation of the current recommendation to introduce antibiotics immediately after the diagnosis of acute appendicitis [24] and to continue the antibiotics in the postoperative setting in case of complicated appendicitis [24].

Furthermore, we observed a relative increase of cases with non-operative management from 9.9 % to 15.9 % during COVID-19 period (p=0.074). These numbers are similar to the results of the recent German meta-analysis (13–16 %), convincingly less than 54 % published in a multicenter cohort study from UK [11, 34]. Consequently, a more often and longer use of antibiotics was necessary for conservative treatment compared to surgical cohort (p<0.001; p=0.02), but it led to shorter overall length of stay and less complications when compared to surgery (p<0.001; p=0.016). The failure of conservative treatment after at least 24 h of treatment is documented in 10.9 % (6 of 55 cases), which matches to reported rates in the literature [12]. Also, the rate of readmissions within first 3 months of follow up was relevantly higher after conservative treatment than after surgery (6.1 % vs. 0.6 %, p=0.001), possibly indicating that a part of surgical patients with low grade complications were treated by family doctors. However, the case series (5 patients) is too low for definitive statement about readmission rate depending on treatment modality.

Lower morbidity and following shorter overall length of stay with shorter antibiotic therapy (p=0.005; p=0.052; p<0.001) related to the entire study collective, implicate better short-term outcome of appendicitis therapy during COVID-19 period in comparison to the collective of non-COVID-19 period in our institution.

The COVID-19 pandemic has changed our daily routine relevantly. The regulations during the first national lock down in Germany following restricted virus transmission policy have been applied in our institution [22]. In contrast to UK reports to SARS-CoV-2 Test rate of 32 % at the beginning of pandemic [11], in our center we required obligatory swab test before admission or entering the operating theatre. This enabled good triage with safe processing of patients, followed by lowering the infection risk for medical stuff and other patients.

There were no COVID-19-positive patients with appendicitis in our investigated collective (only 3 children, that were excluded), whereas only 0.3 % of suspected SARS-CoV-2 infections among appendicitis patients were tested positive according to other available data [32].

Regarding the time spent in the emergency room, we interestingly recorded shorter waiting times during COVID-19 period than before pandemic for over 2 h per admission. A remarkable decrease in emergency room cases during the COVID-19 pandemic was reported in some recent studies, as eventual explanation for this observation [35]. On the other hand, a strict organization and patient triage might have leaded to faster transfer to corresponding ward or discharge.

According to our analysis the overall number of appendicitis cases among adults in our institution has decreased during the COVID-19-pandemic by 10.35 % and the number of appendectomies by 16.4 %. The latest meta-analysis from Germany reported a decrease in overall appendicitis case number of 20 % and reduction of appendectomies of 12.9 %, similar to our findings [34]. However, when compared to this data reported, during the pandemic there was a lower reduction of appendicitis cases overall, but a higher reduction of appendectomies. The last one indicates a relevant increase in conservative treatment.

## Limitations

The present study is a retrospective single-center study, with limited case number and inclusion of only adult patients. We are aware, that the present study includes low number of cases with conservative treatment for adequate comparison with outcomes of operative treatment and a short-term follow up. Furthermore, there was no randomization in terms of treatment choice.

## Conclusions

The COVID-19 pandemic had a relevant impact on management of acute appendicitis at our institution, leading to reduction of appendectomy rate. The present study demonstrates that by maintaining the system of emergency treatment and performing a good case triage, the quality of treatment and outcome can be kept at a very good level, even under pandemic circumstances. Additionally, the implementation of a sufficient screening system before admission is essential to avoid SARS-CoV-2 infections under patients or hospital-staff. Furthermore, the increase of laparoscopic approach for appendicectomy and selective, but critical use of antibiotics leads to lower postoperative morbidity. The pandemic also highlighted the potential of conservative treatment for well selected cases, ensuring shorter length of stay and morbidity than after surgery in short term outcome.

Laparoscopic appendicectomy remains the standard procedure for treatment of acute appendicitis in Germany with potential for improvement of outcomes even throughout pandemic conditions. Non-operative management shows excellent short-term outcome and can be safely applied for uncomplicated appendicitis as an alternative to surgery during potential new waves of COVID-19 pandemic or any other conditions, where surgical treatment cannot be immediately provided. Of course, further evidence in terms of RCT in patients with uncomplicated appendicitis is needed, in order to consider conservative treatment as a standard procedure in this patients’ collective.

## Supplementary Material

Supplementary Material
