# Supplementary material for: Management of acute appendicitis during COVID-19 pandemic. Single center data from a tertiary care hospital in Germany
Source: Innov Surg Sci. 2023 Nov 13;8(2):39–48. doi: 10.1515/iss-2022-0021 (PMC10696938; doi:10.1515/iss-2022-0021)
Supplement: Supplementary file 1 — Supplementary Material [file j_iss-2022-0021_suppl_001.pdf]

Reviewer Assessment

M. Andric et al.: Management of acute appendicitis during COVID-19 pandemic. Single center data from a tertiary care hospital in Germany

Reviewers' Comments to Original Submission

Reviewer 1: anonymous

Date received: 21-Feb-2023
Reviewer recommendation: **Accept in present form**
Reviewer overall scoring: **High**

Comments to author:
This is a nicely presented study of the diagnosis and treatment of acute appendicitis during the Covid-19 pandemic. The study has some limitations - single-center, retrospective, small numbers of conservatively treated patients, not really new data -, but these limitations are clearly named by the authors. Despite these limitations, the conclusions appear to be justified. The manuscript should be published in present form..

Reviewer 2: anonymous

Date received: 02-Apr-2023
Reviewer recommendation: **Accept in present form**
Reviewer overall scoring: **High**

Comments to author:
The presented data show a change of treatment in (uncomplicated) acute appendicitis during COVID-19 pandemic to more conservative procedure with good short-term outcome. Referring to diverse studies in terms of non-operative concepts the conservative therapy can be safe performed as far as a appropriate selection is implemented.
